# How expensive is a healthy diet in Europe? Using Linear Programming as a standardised method for calculating European Food Reference Budgets

**DOI:** 10.1017/S1368980025101316

**Published:** 2025-11-03

**Authors:** Mareike Taeger, Silke Thiele

**Affiliations:** 1 ife Institute of Nutrition and Food Economics, Fraunhoferstraße 13, 24118 Kiel, Germany; 2 Department of Food Economics and Consumption Studies, University of Kiel, Olshausenstraße 40, Kiel 24118, Germany

**Keywords:** Reference Budgets, Cost of a healthy diet, Food insecurity, Linear Programming

## Abstract

**Objective:**

According to the principles of the European Pillar of Social Rights, everyone should be entitled to an adequate minimum income sufficient for a healthy diet. Studies show that food insecurity remains a concern in Europe, highlighting the need to determine budgets for adequate nutrition, known as Food Reference Budgets. Previous approaches, based on expert-designed food baskets or focus group discussions, are often limited by their normative nature and/or low representativeness.

**Design:**

To address these problems, this study explores Linear Programming as a novel method to calculate Food Reference Budgets for twenty-six EU member states. To analyse if Linear Programming could be an adequate tool to calculate future Food Reference Budgets, this method was used to design country-specific food baskets that align with consumption habits and healthy diet requirements. The food baskets were then priced at different levels to determine the cost for healthy diets in twenty-six European countries.

**Setting:**

Germany.

**Participants:**

The calculations are based on consumption data from the EU Household Budget Survey (EU-HBS) from 2015 (2010 for Malta, Portugal and Slovenia). For Germany, data from the national income and consumption survey was used instead, as German data was not included in the EU-HBS.

**Results:**

The results show a positive correlation between optimised healthy and current observed diets for most food groups, indicating that country-specific preferences are reflected in the optimised healthy food baskets. Nevertheless, to meet healthy diet requirements, consumption of vegetables, fruit, fish and dairy must increase compared with the current observed diets. At a lower price level, the Food Reference Budgets ranged from 2·38 to 5·71 €/day, depending on the country. With a low-price level (20th percentile), costs for healthy diet accounted for between 5·74 % of income in Luxembourg and 29·00 % in Romania, showing the large differences in affordability between countries.

**Conclusion:**

Overall, it was concluded that Linear Programming could be a promising approach for determining uniform and comparable European Food Reference Budgets and should be discussed in the context of the EU Commission’s efforts to modernise the European minimum income schemes.

According to the principles of the European Pillar of Social Rights, everyone should be entitled to an adequate minimum income benefit that enables a life in dignity^(1)^. An essential basis for a dignified life is a sufficient supply with healthy food. However, studies have shown that people in Europe are still affected by food insecurity^(2,3)^, which means that they do not have ‘access to sufficient, safe and nutritious food to meet their dietary needs and food preferences for an active and healthy life’^(4)^. A key reason for limited access is lack of financial resources^(5)^. In fact, in Europe, at least 10 % of the population in sixteen out of twenty-four countries cannot afford a healthy diet due to insufficient income^(3)^. Given the central role of financial resources in enabling access to healthy food, identifying the minimum necessary budget is an important step in combating food insecurity.

In order to determine the level of income that is at least necessary to be sufficiently supplied with healthy food, Reference Budgets are considered as an appropriate tool^(6)^. Reference Budgets are price-valued baskets of goods (here food baskets), which have previously been calculated according to three different methods which include (1) public-led-, (2) expert-led- and (3) survey-led approaches^(7)^. These methods are sometimes also used in parallel, that is, complementing each other. While the (1) public-led approach brings citizens together to design and discuss adequate baskets of goods and prices (focus-group discussion), the (2) expert-led approach involves experts who design the food baskets based on recommendations and guidelines. On the other hand, the (3) survey-led approach is based on the analysis of survey data from which the food baskets are derived^(7)^.

Previous approaches calculated Food Reference Budgets mainly based on the (1) public- and (2) expert-led approaches^(e.g.(8–12))^. However, due to their normative character and low representativeness, these methods are often criticised in the literature. Against this background, this study aims to calculate Food Reference Budgets for the member states of the EU using a survey-led approach. Building on the method that we have already used in the project ‘Measuring and monitoring absolute poverty (ABSPO)’ by Menyhért *et al.* 2021^(13)^, we calculate Food Reference Budgets using Linear Programming, which can be characterised as a form of the survey-led approach. Compared with the calculations used in the ASPBO, we have made a number of adjustments to the calculations in this study (taking additional nutrients into account, e.g. cholesterol, using new price data). The results are therefore not directly comparable.

With Linear Programming, food quantities can be identified that meet all requirements of a healthy diet, such as covering nutrient needs, while deviating as little as possible from observed consumption habits of the population. The use of the Linear Programming method as a basis for calculating Food Reference Budgets was already proposed by Godemé *et al.* ^(11)^.

Against this background, the aim of this study is to analyse if the Linear Programming method could be an adequate approach to calculate future Food Reference Budgets. To pursue this goal, this study proceeds as follows: After discussing the different methods underlying previous Food Reference Budgets calculations, the Linear Programming method is described and used to identify healthy food baskets for twenty-six European countries, considering the country specific consumption habits. Based on correlation analyses it is examined if the identified optimised healthy food baskets reflect the consumption patterns of the individual countries. In a further step, the optimised healthy food baskets are priced to calculate country specific Food Reference Budgets. For this purpose, consumer price index data are used. In order to check the plausibility of the results on a case study, current Food Reference Budgets applied in Germany are compared with those calculated in this study.

## Methods and data

### Overview of methods for calculating Food Reference Budgets

When determining food baskets as the basis for Food Reference Budgets, several requirements should be met: the baskets should be healthy and socially acceptable. In addition, they should also be accessible and affordable. The requirements of health and social acceptance in particular often cause conflicts because current eating habits usually deviate from dietary recommendations. Finding healthy food baskets that are socially accepted is therefore a particular challenge and is solved differently in the various approaches to determining Food Reference Budgets.

#### Expert- and public-led approaches

In order to address the two aspects ‘health’ and ‘social acceptance’ simultaneously, the expert- and public-led approaches make use of nutrition experts and focus groups. In the expert-led approach, nutrition experts compile healthy food baskets based on dietary recommendations. Where appropriate, the acceptability of the food baskets is verified through focus group discussions^(e.g.(8–12))^. In the public-led approach, the process is in reverse order. First, menu plans are created through focus groups. Then, these are adjusted by nutrition experts for their compliance with the dietary recommendations^(e.g.(14,15))^.

The expert-led approach was applied, for example, in a pilot project called ImPRovE-Approach, where European Reference Budgets were calculated^(11)^, that is, in a first step nutrition experts complied food baskets, which were adjusted by focus groups in a second step. Although the approach of this project was generally considered promising, some limitations related to the expert-led approach and focus group discussions were mentioned in the literature. The composition of food baskets by experts is generally criticised for its normative character, as it could be influenced by the experts’ attitudes and opinions^(16)^. This is particularly critical because experts usually have a higher education and income and therefore may have a different perception of needs^(16)^. In the ImPRovE-project, the dietary recommendations used by experts also varied widely between countries, making it difficult to compare results across countries^(11)^.

Focus group discussions are considered as an important tool for determining a socially acceptable minimum standards of living, as members of society determine what is essential for life. However, as a main limitation of the focus group discussions their low representativeness is mentioned^(17)^. Due to the limited number of people participating in the focus groups, the results do not necessarily reflect general public opinion^(17)^ and therefore have a low robustness^(18)^. Furthermore, it is difficult to find a common consensus in focus group discussions^(19)^. This was shown, for example, by a Dutch study in which Reference Budgets differed considerably. For a couple with children, the focus groups calculated for the food sector monthly budgets between €491 and €730^(20)^.

#### Survey-led approaches

While the previously described approaches, when used in combination, consider both aspects ‘health’ and ‘public acceptance’, the survey-led approach usually focusses on the second aspect. Here, survey data are used to investigate households’ consumption patterns either in terms of quantities or expenditures. Often, expenditure data of low-income households are used to directly derive Reference Budgets. However, this approach is criticised because of its circularity: As the expenditure behaviour of low-income households is probably influenced by budget restrictions, an adequate living standard cannot be guaranteed^(7)^. This is also of particular relevance for food, as it has been empirical observed that an unhealthier diet is associated with lower costs^(21–24)^ and is increasingly chosen by households with lower socio-economic status^(23)^. Another limitation of expenditure data is that it does not account for the variability in prices arising from differences in brand, quality or point of sale (e.g. discount supermarket *v*. specialty store). Therefore, when deriving a Food Reference Budget based on the food expenditure of lower income groups, it cannot be guaranteed that the budget for a healthy diet is sufficiently high.

#### Linear programming – a survey-led approach

Against the background of the limitations of the methods used so far, Godemé *et al.* proposed to apply the Linear Programming (LP) method for the determination of Food Reference Budgets^(11)^. LP is a scientifically recognised method that has already been used in international nutritional studies ^(e.g.(25–28))^. However, it has not yet been applied in the context of determining Reference Budgets. LP can be characterised as a form of the survey led approach, as in a first step, representative food consumption patterns can be derived from survey data, which in a second step can be adjusted using LP to integrate the health aspect. With LP, it is possible to transform an observed diet into a healthy one (for developing healthy food baskets) in such a way that the deviation from the current observed consumption is minimised while complying with restrictions (e.g. nutrient guidelines). Thus, the optimised food baskets are healthy and as close as possible to the observed consumption. After optimisation the healthy food baskets can be evaluated with prices to determine the Food Reference Budgets. In this study, we want to use LP for the determination of Food Reference Budgets. As a basis for the calculations, we need various datasets that are described below.

### Data

To determine healthy food baskets for twenty-six European countries, considering country-specific consumption habits, two datasets were needed: (1) consumption survey data from all EU countries to identify the consumption patterns and (2) nutrient data to provide information on the nutrients contained in the foods. To calculate Food Reference Budgets (3), price data for all EU countries are needed in addition.

#### Consumption survey data

To identify the country-specific food consumption patterns, we made use of the EU Household Budget Survey (EU-HSB). This dataset contains information on expenditures and quantities and the foods groups are recorded in the European classification of consumption by purpose (ECOICOP). In the detailed 5-digit ECOICOP classification, the categorisation comprises sixty-two food groups, which are predominantly commodity based. Data on expenditure and quantities for out-of-home consumption (e.g. in restaurants or canteens) were not available. The dataset therefore reflects food consumption at home. The EU-HBS are compiled every 5 years based on national HBS surveys. These include one or more interviews/diaries depending on the country. Detailed information on the survey can be found in an Eurostat-report^(29)^. The data used in this study are from 2015 (or 2010 for Malta, Portugal and Slovenia).

As consumption data for Germany were not included in the EU-HSB, the German Income and Consumption Survey (EVS) was used in addition. The EVS is part of the official statistics and is conducted every 5 years as a household survey. The participating households are selected based on a quota plan. Part of the overall survey consists of a survey on the purchases of food, beverages and tobacco products, in which households record their purchased quantities and expenditures over a 1-month period. At the time of the study’s preparation, the most recent survey available was the one from 2013 which covered 11 416 households with different household sizes and compositions. However, as nutrient and food recommendations (here European Food Safety Authority (EFSA) recommendations) are only given for single individuals, we made use of the OECD-modified equivalence scale to derive individual consumption quantities from the household consumption. Therefore, total household food quantities were divided by the respective weights of the equivalence scale.

While food and beverages are classified into 206 different groups in the German EVS consumption data, they were divided into sixty-two groups in the European Household Budget Survey. These 62 groups correspond to the 5-digit ECOICOP which is a commonly known classification system developed by the United Nations Statistics Division^(30)^. In order to standardise the consumption datasets (EU-HBS and EVS), the 206 food and beverage groups of the German EVS were assigned to the sixty-two ECOICOP groups. However, since there were no foods in the EVS that could be assigned to the ECOICOP group ‘other tubers/ products of tuber vegetables’, consumption data were only available for 61 of the 62 ECOICOP groups for Germany. Furthermore, as two of the sixty-two ECOICOP groups, namely alcoholic beverages and baby food, were not of interest for the calculation of Food Reference Budgets, they were excluded from the analysis. In the end, the calculations were based on fifty-nine food groups for Germany and sixty for the other EU countries.

#### Nutrient data

To obtain information on nutrient contents, the German food composition database (Bundeslebensmittelschlüssel Version 3·01, BLS), which gives information on nutritional values for 14 814 foods available in the German market, was used. Each of the sixty food groups was assigned to the corresponding food groups of the nutrient database. If a clear assignment to a single food group was not possible, the average nutrient values of two or more food groups were used.

#### Price data

To price the food basket for Germany, data collected for the calculation of the German Consumer Price Index 2018 were used. The prices of the German Consumer Price Index are collected monthly in supermarkets and discounters throughout the country. Over the year 2018, a total of 1 199 968 individual food prices were gathered, which are summarised by the Federal Statistical Office in 170 groups. These 170 groups were assigned by us to the sixty ECOICOP groups. For each ECOICOP group, an average price per kilogram was calculated based on the available German Consumer Price Index prices of the assigned items. As prices within food groups can vary depending on factors such as brand or place of purchase (e.g. discount stores *v*. supermarkets), we calculated the Food Reference Budgets using several lower price percentiles (20th, 30th, 40th and 50th) to capture a realistic range of possible food costs.

In order to also get prices for the other EU countries Eurostat-Price level indices from 2018 were used. The price level indices reflect the relative price level of countries in relation to another country. The indices are subdivided in the ECOICOP 5-digit classification and were therefore directly available in the classification required for this study. The price level indices are available for all EU countries except Croatia. Therefore, the calculation of Food Reference Budgets based on the optimised food baskets could be calculated for twenty-five instead of twenty-six countries.

#### Income data

To assess the affordability of healthy food in each country, we determined the cost of the healthy optimised food baskets as a proportion of income. Specifically, we used the median net equivalised income for the 25–54 age group provided by Eurostat (Eurostat 2022)^(31)^. This indicator enables a cross-country comparison of disposable household income. By relating the cost of optimised healthy food baskets to income, we were able to assess the relative financial accessibility of a healthy diet.

### Empirical application of the linear programming model

The LP model includes an objective function, decision variables and restrictions. The formulation of these components in application to our research question is described below.

#### Objective function

The objective function of the LP includes decision variables whose values are the outcome of the model. In this study, the outcome are optimal food quantities resulting from a minimisation of deviation to a current observed diet (objective function) while meeting constraints such as nutrient recommendations. The linear objective function *Y* has the form 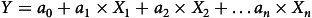 , *where X*_*1*_ *, …X*_*n*_, are the decision variables and *a*_*0*_ *, …a*_*n*_ are constant.

The aim in this study is to build the objective function in such a way that it minimises the absolute deviation between a current observed diet and the optimised healthy diet calculated by the LP. As a starting point for optimisation, first the current observed mean consumption *C*_*i*_ (with *i* = 1 to *n*, where *n* is the number of food groups) was calculated based on the consumption data. However, a calculation of the total consumed energy content of the food groups showed that the amount of energy in some countries deviates significantly from the recommendations. Therefore, the percentage energy structure between the food groups was used to proportionally adjust the food quantities so that the energy content of the total food consumption corresponds to the EFSA recommended calorie intake. This energy scaled consumption (*sC*_*i*_) represents the countries’ current observed diet at the level of the recommended energy intake and is the starting point for optimisation. The scaled consumption is defined as follows:

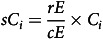




*C*_*i*_ observed mean consumption of *i*

*sC*_*i*_ scaled mean consumption of *i*

*rE* recommend energy intake

*cE* observed mean energy intake of a country

with *i* = 1 to *n*, where *n* is the number of food groups

The absolute deviation is defined as the difference between the quantities of each food item *X*_*i*_ calculated by the LP (with *i = 1…n*, where *n* is the number of food groups) and the observed scaled consumption *sC*_*i*_, divided by *sC*_*i*_. Dividing by the quantity *sC*_*i*_ aims to standardise the differences between the food groups. The approach follows Darmon *et al.* ^(26)^ and is based on the principle of the goal programming approach^(32)^. The function of the ‘total departure of mean food intake’ is thus as follows:

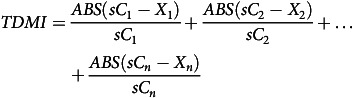




*TDMI* is the total departure of mean food intake

*X*_*i*_ decision variable: optimised amount of *i*

*ABS* absolute value

By introducing further decision variables and restrictions, this non-linear objective function can be transformed into a linear objective function of *X*_*n*_^(described in detail in^ ^(26))^. With the help of this transformation, the function can be solved as a Linear Programming problem using OpenSolver in Excel.

#### Restrictions

The restrictions used in this study are divided into two parts. On the one hand, health-related restrictions on nutrient and food recommendations were considered to model a healthy diet. On the other hand, consumption restrictions were included to ensure that all food groups consumed by the population, including those considered rather unhealthy, such as sweets, are included in the optimised diet.

##### Health-related-restrictions

The food baskets in this study were calculated for adult men and women with medium activity levels. To account for nutrient-related constrains, the reference values for adequate nutrient intake of the EFSA were chosen, using a physical activity level of 1·4 corresponding to sedentary work with little or no strenuous leisure activities^(33)^. To ensure age-appropriate nutrient intake for adults, the reference values for 25–49-year-olds were used. In addition to lower limits, the EFSA also gives upper limits for some nutrients that should only be consumed to a limited extent^(34)^. The lower and upper limits used as restrictions in this study are listed in Table 1.

**Table 1.** Nutrition-related constraints used in the linear programming model

	Unit/day	Quantities per day	
		Male	Female
Energy	MJ	9·4	7·55
Dietary fibre	g	25	25
Total carbohydrates^*^	% of energy intake	45–60	45–60
Total carbohydrates^*^	g	252·6–336·8	202·9–270·5
Alpha-linolenic acid (ALA)^*^	% of energy intake	0·5	0·5
Alpha-linolenic acid (ALA) ^*^	g	1·2	1·0
Eicosapentaenoic acid, DHA (EPA, DHA)	mg	250	250
Linoleic acid (LA)^*^	% of energy intake	4	4
Linoleic acid (LA)^*^	g	10·0	8·0
Total fat^*^	% of energy intake	20–35	20–35
Total fat^*^	g	49·9–87·3	40·1–70·1
Protein^*^	g/kg bodyweight	0·83	0·83
Protein^*^	g	68·1	54·8
Saturated fat^*^	% of energy intake	10	10
Saturated fat^*^	g	24·9	20·0
Cholesterol	mg	300	300
Calcium	mg	950–2500	950–2500
Chloride	g	3·1	3·1
Copper	mg	1·6–5	1·3–5
Iodine	µg	150–600	150–600
Iron	mg	11	16
Magnesium	mg	350	300
Manganese	mg	3	3
Molybdenum	µg	65	65
Phosphorus	mg	550	550
Potassium	mg	3500	3500
Selenium	µg	70	70
Sodium	g	2	2
Zinc	mg	12·85–25	10·125–25
Biotin (B7)	µg	40	40
Choline	mg	400	400
Cobalamin (vitamin B12)	µg	4	4
Folate (B9)	µg DFE	330	330
Niacin (B3)^*^	mg NE/MJ	1·6	1·6
Niacin (B3)^*^	mg	15·0	12·1
Pantothenic acid (B5)	mg	5	5
Riboflavin (B2)	mg	1·6	1·6
Thiamine (B1)^*^	mg/MJ	0·1	0·1
Thiamine (B1)^*^	mg	0·9	0·8
Vitamin A	µg	750–3000	650–3000
Vitamin B6	mg	1·7–25	1·6–25
Vitamin C	mg	110	95
Vitamin D^†^	µg	5–100	5–100
Vitamin E as *α*-tocopherol	mg	13–300	11–300
Vitamin K as phylloquinone	µg	70	70

Source for lower and upper recommendation:^(33)^ and^(34)^.

Source Cholesterol:^(35)^.

* Recommmendation made in % of energy intake, in g/kg bodyweight or in mg/MJ are converted in g or mg considering the following assumptions: body weight: 82 kg (man), 66 kg (women)^(36)^; PAL: 1·4 (man and woman); energy intake: 9·4 MJ (man), 7·55 MJ (woman)^(33)^; Energy contents of energy-providing nutrients according to EC Directive (90/496/EEC) were used.

† As Vitamin D can be ingested through food as well as produced by the body through endogenous synthesis a lower limit of 5 µg per day was used.

In addition to the reference values for nutrient intake, there are also some food-related recommendations that are important for a healthy diet. This applies to fruits, vegetables, water, salt, sugar and caffeine. The food-related recommendations used in this study, their sources and more detailed explanations can be found in Table 2.

**Table 2.** Food-related constraints used in the linear programming model

	Quantities per day	
	Male	Female
Vegetable^*^	294·7 g–589·3 g	236·7 g–473·3 g
Fruits^*^	196·4 g–392·9 g	157·8 g–315·6 g
Salt**^†^**	6 g	6 g
Sugar **^‡^**	56·1 g	45·1 g
Water**^§^**	2500 ml–3250 ml	2000 ml–2600 ml
Coffee and Tea (Caffeine) **^||^**	800 ml	643·9 ml

* Source: The recommendation that fruits and vegetables should provide between 7 and 14 % of energy intake^(37)^ were converted in g/d. For this following information were used: Recommendation that fruits and vegetables should be consumed in the following proportions: 2 portions of fruits and 3 portions of vegetables^(38)^; Average calorie content of fruits/vegetable: 50 kcal/ 20 kcal^(37)^, which, considering the recommended proportions, corresponds to an average caloric content of 32 kcal for vegetables and fruits.

† Source:^(39)^.

‡ Source: The recommendation of reducing the intake of free sugars to less than 10 % of total energy intake^(40)^ were converted in g.

§ Source: The recommendation includes water from beverages and from food^(33)^. To avoid beverages being represented in disproportionate amounts, an upper limit was set that allows for a maximum deviation of 30 % above the recommended water amount.

|| Source: As caffeine is not included in the nutrient database, the limitation of caffeine intake had to be done by limiting the amounts of coffee and tea. Up to 400 mg of caffeine per day is regarded as safe^(41)^. With a caffeine content of less than 100 mg per cup of coffee (200 ml)^(41)^, 4 cups are within the safe intake level. Since the recommendations for caffeine intake are based on body weight, the limit for women was scaled accordingly based on body weight.

##### Consumption-related restrictions

In order to ensure that all food groups consumed by the population are represented in the food basket, lower consumption limits were chosen for each food group. It was assumed that the quantity of all food groups must be at least 10 % of the mean observed diet. A higher lower limit (e.g. 20 %) could not be chosen because many of the calculated models were then not solvable. This is because the diet observed in the population tends to be unhealthier (too fatty, too salty, too sugary, etc.) and therefore requires a larger deviation from existing consumption patterns, for example, in terms of sweets and snacks, to meet nutrient requirements. In the Czech Republic, the model was not solvable even with the 10 % lower limit. Therefore, the limit was lowered in 1 % steps until the model could be solved at the 8 % limit.

## Results

### Developing country-specific healthy food baskets

With the help of LP, we calculated healthy food baskets for twenty-six European countries that deviate as little as possible from the current observed diets and met the recommended intake levels for all considered macro- and micronutrients in accordance with EFSA nutrient reference values. Table 3 shows the percentage changes between current observed and optimised healthy diets on average for all countries for ten individual food groups. The values reveal that significant changes in dietary habits would be required to achieve the nutrient- and food-related recommendations for a healthy diet. For example, the quantity of vegetables would have to increase by 230·9 % for men and 278·6% for women. Further significant increases of more than 100 % would be required for fruits, fish and dairy products. In addition, women would have to increase their consumption of eggs by 164·9%. As expected, however, the consumption of other foods would have to be reduced in the same course. This applies in particular to the food groups sweets and snacks and fats and oils. In addition, the mean quantities of the food groups in the optimised diets are shown in Table 3. It becomes clear that, for example, comparatively large quantities of vegetables would have to be consumed. They are 521·6 g per day for men and 472 g per day for women. In contrast, the consumption of sweets and snacks is only 33·7 g per day for men and 12·9 g per day for women.

**Table 3.** Percentage changes between observed and optimised diets and quantities in optimised diets for food groups on average across twenty-six European countries

	Mean changes [%]^*^		Quantity of optimised diet (g/d)	
	Man	Woman	Man	Woman
Foods rich in carbohydrates	6·2	1·2	322·0	243·9
Fruits	134·0	138·7	331·8	270·9
Vegetables	230·9	278·6	521·6	472·0
Milk & Dairy Products	100·8	159·2	428·9	434·0
Meat	25·0	19·3	157·8	114·5
Fish	137·9	210·6	49·2	55·1
Egg	25·3	164·9	20·3	33·7
Sweets & Snacks	−35·0	−68·3	33·7	12·9
Oils & Fats	−52·6	−68·8	15·5	8·6
Others **^†^**	−51·4	−20·2	21·2	25·6
Total quantity of food	62·1	77·9	1901·9	1671·2
Total quantity of beverages	132·5	169·0	1121·1	1046·1

* First, the percentage changes of a group (e.g. for fruits) were calculated for all countries individually. Then, a mean value of the percentage change was calculated for each group.

† The ‘others’ group is composed of sauces, salt, spices and ready-made meals.

**Figure 1:**
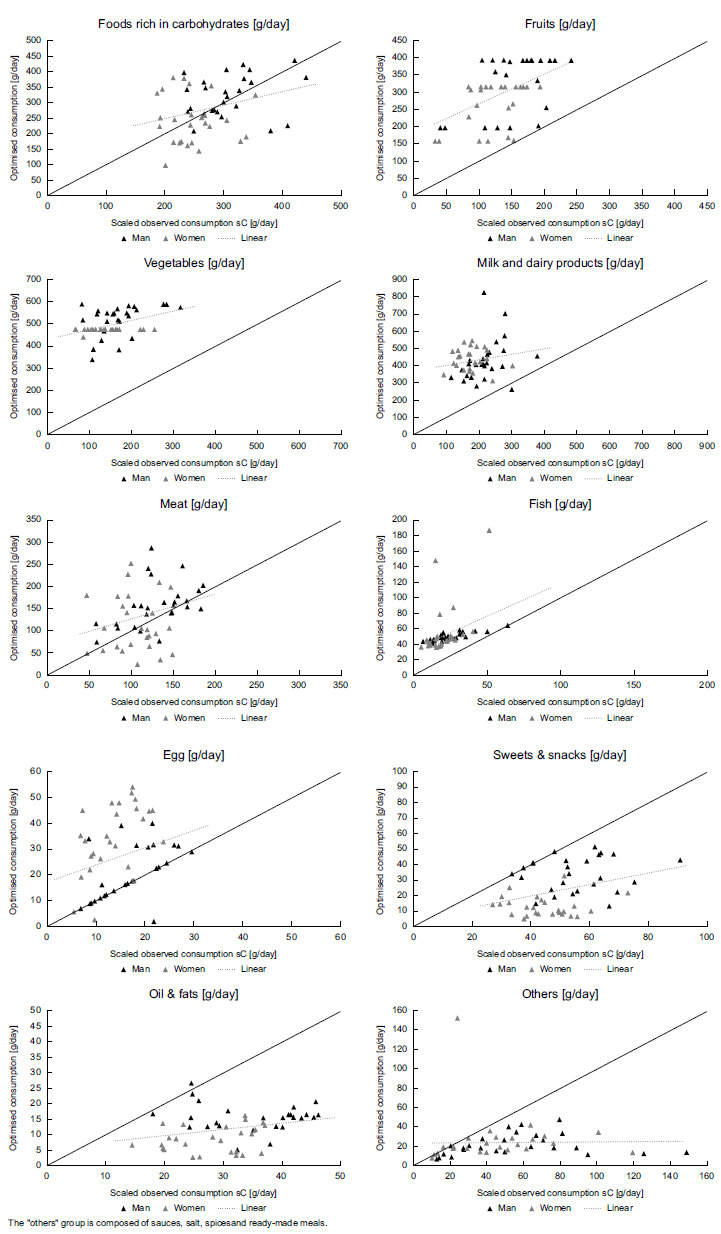
Quantities of observed and optimised diets for food groups for 26 European countries differentiated by gender. The ‘others’ group is composed of sauces, salt, spices and ready-made meals.

Whereas Table 3 refers to mean changes across the countries the following graphics show how much the changes are distributed between the countries. Figure 1 illustrates the quantitative changes between scaled observed (*sC*_*i*_) and optimised healthy (*X*_*i*_) diets for the twenty-six European countries differentiated by gender. If the spots are above the angle bisector, the amounts in the optimised healthy diet are higher than in the current observed consumption. If the spots are below the angle bisector, the amounts in the optimised healthy diet are lower. It can be seen that the observations in the two categories foods rich in carbohydrates and meat are distributed around the bisector, that is, to meet all nutrient- and food-related recommendations some countries would have to reduce their consumption of these food groups, while others would have to increase it. In contrast, in the categories fruits, vegetables, milk and dairy products and fish all countries are above the bisector and would therefore have to increase the consumption of these food groups without exception. Since the observations in the categories sweets and snacks, oils and fats and others are almost all below the bisector, consumption would have to be reduced.

Figure 1 already indicated the presence of positive correlations between current observed and optimised healthy diets. The level of the correlations and their statistical significance is shown in Table 4. It is also evident here that the correlations are positive and except for the categories milk and dairy products and others they are statistically significant. Overall, the results indicate that country-specific dietary preferences are reflected in the optimised healthy food baskets because comparatively low consumption quantities in the current observed diets are accompanied with comparatively low quantities in the optimised healthy diets and vice versa.

**Table 4.** Correlations between observed and optimised consumption for food groups on average across 26 European countries

	Both sexes	
	Corr.coeff.	*P*-value
Food rich in carbohydrates	0·314*	0·023
Fruits	0·499**	0·000
Vegetables	0·419**	0·002
Milk & Dairy Products	0·177	0·210
Meat	0·313*	0·024
Fish	0·399**	0·003
Egg	0·287*	0·039
Sweet & Snacks	0·354*	0·010
Oils & Fats	0·292*	0·036
Others	0·021	0·883
Total amount of food	0·686**	0·000
Total amount of beverages	0·489**	0·000

*Significance level 5 %; **Significance level 1 %.

### Calculating Food Reference Budgets

**Figure 2:**
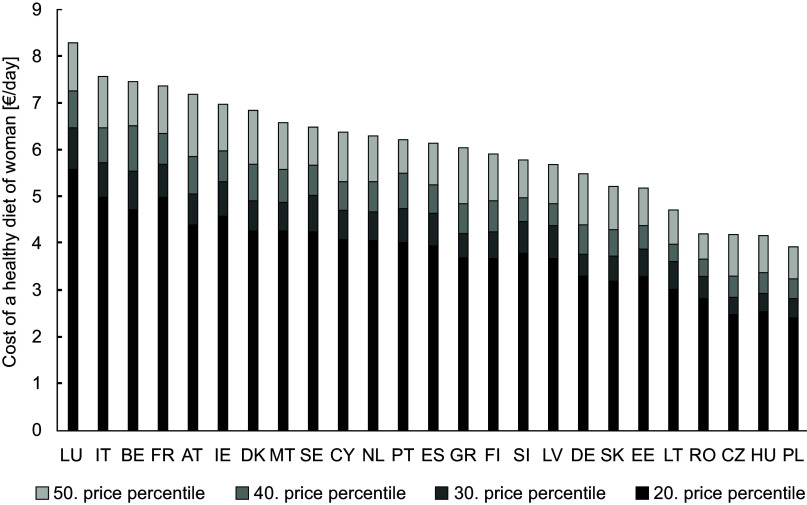
Cost of a healthy diet for women in 25 European countries (without Croatia).

**Figure 3:**
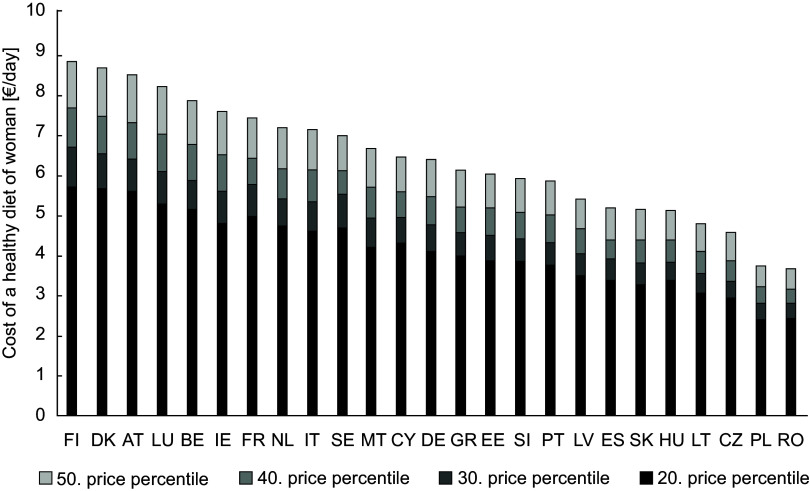
Cost of a healthy diet for men in 25 European countries (without Croatia).

In a next step, the optimised food baskets were priced to determine the costs of healthy diets per day. As the focus is on minimum costs, the valuation of the food baskets should be based on comparatively low prices. Therefore, a range of four different lower price percentiles were considered: the 20th, 30th, 40th and 50th. Figures 2 and 3 show the food costs per day for the optimised diets in the different countries differentiated by gender. While for men the cost of a healthy diet at the 20th price percentile ranges between 2·38 €/day (Poland) and 5·71 €/day (Finland), for women the range is from 2·40€/day (Poland) to 5·57 €/day (Luxemburg). By purchasing food at the 50th price percentile, the cost increase for man up to 3·66 (Rumania) and 8·84 €/day (Finland) and for women up to 3·94 (Poland) and 8·28 €/day (Luxemburg).

In Germany, €4·75 per day is the nationally standardised amount allocated specifically for food within the basic social benefit scheme. The benefit is structured by expenditure categories (e.g. food, clothing and housing) and does not vary across regions^(42,43)^. According to the results of this study, the Food Reference Budgets for a healthy diet in Germany should have ranged between 4·10 and 6·40 € for men and 3·30 and 5·50 € for women. This means, if Germans bought food in a price-conscious way in 2018, they could have financed a healthy diet with their social benefits. More precisely, social benefits in Germany in 2018 enabled the purchase of a healthy diet at the 30th price percentile for men and at the 40th price percentile for women. Although the total benefit is paid as a lump sum and may also be used for other purposes, the 4·75 € represent the officially allocated amount for food and thus provide a relevant benchmark for assessing the adequacy of minimum income provisions in relation to a healthy diet.

**Figure 4:**
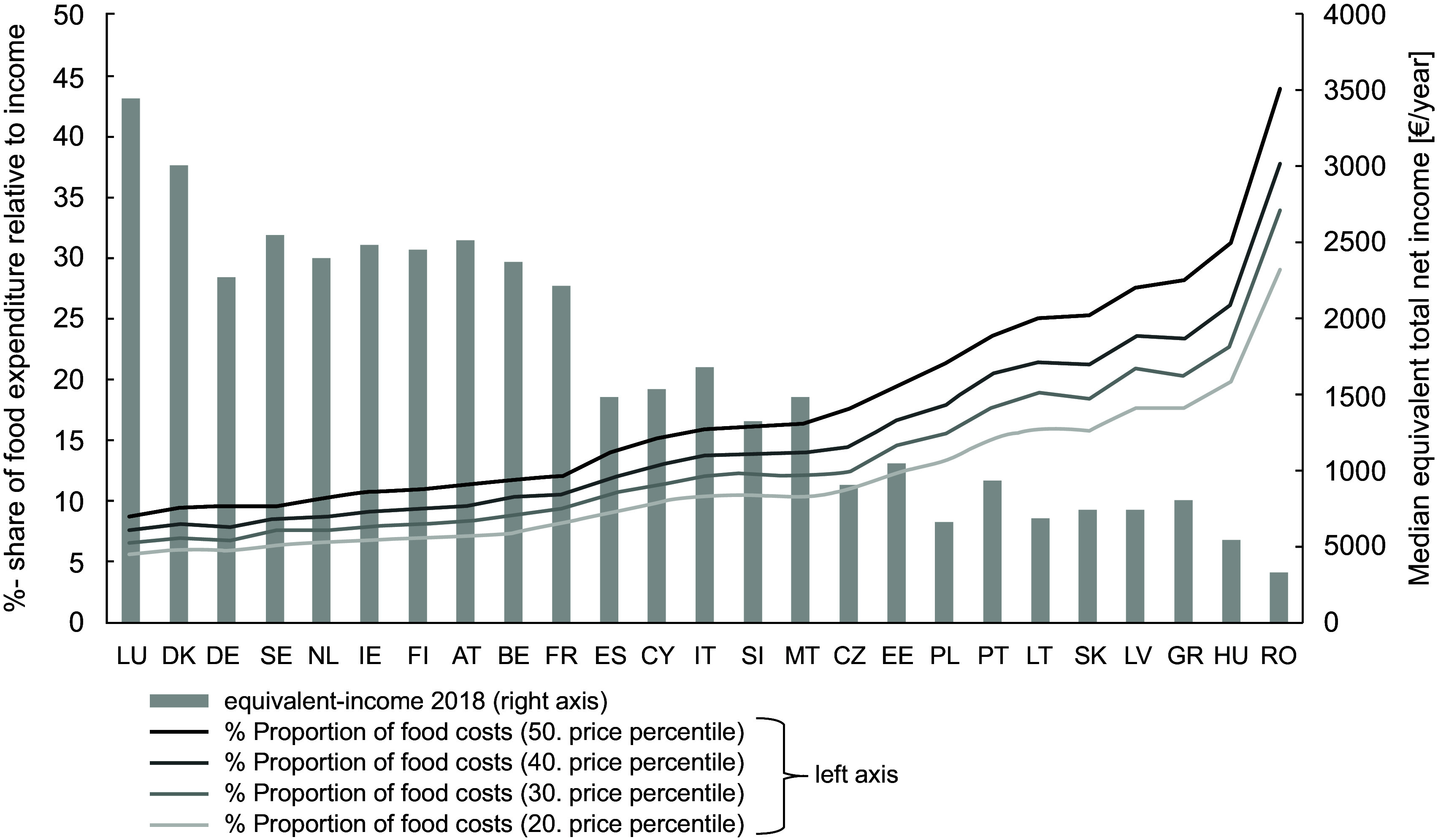
%-shares of costs of optimised food baskets averaged for men and women (for price percentiles 20–50) in the median equivalised total net income for the age group 25–54.

In order to assess the level of the calculated costs for all included EU countries, their percentage share in income (left axis) and the median equivalised net income (right axis) is shown in Figure 4. For the calculation of the percentage share the median net equivalised income for the age group 25–54, which is given for both sexes together, was used^(31)^. Figures 2 and 3 have already shown that the Food Reference Budgets differ between the countries (evaluated with the 20th price percentile, the costs ranged between 2·38 and 5·71 €/day). However, the differences are smaller than the income differences between the countries (net income between 3284 and 34 472 €/year). This is reflected in the very different expenditure shares for a healthy diet in the countries (Figure 4). Evaluated with the 20th price percentile, they are between 5·74 % in Luxembourg and 29·00 % in Romania. Taking the 50th percentile, these shares increase to 8·74 % and 43·76 %. At this percentile, countries such as Luxembourg, Denmark, Germany and Sweden would have to spend less than 10 % of their income on a healthy diet, while countries such as Poland, Portugal, Lithuania, Slovakia, Latvia and Greece would have to spend more than 20 % of their income. As the share of food in household expenditure is considered a poverty measure, these different percentages reflect the wide range of standards of living in the European countries.

## Discussion and policy implications

In this study, the Linear Programming method for calculating Food Reference Budgets was presented and applied. In this framework, the cost of a healthy diet was calculated for twenty-five EU countries to determine the amount of income required for a healthy diet. This is particularly relevant for the social dimension of the EU, which the European institutions are increasingly working to strengthen. This commitment was given particular expression with the twenty key principles of the European Pillar of Social Rights and its action plan which contains concrete measures to put the principles into practise by 2030^(44)^. In this plan, the commission calls on Member States to modernise their minimum income schemes. In order to determine the level of income support, the commission recommended that a method be developed which is (i) transparent and robust, (ii) by which the support can be regularly reviewed and adjusted if necessary and (iii) which offers the possibility of taking individual conditions such as gender and age into account^(45)^. In our study, we presented an approach as a basis for determining minimum income benefits in the food area, which can meet all these requirements.

The approach was Linear Programming that we used for determining the minimum income for sufficient access to healthy food (Food Reference Budgets). First, we identified country-specific healthy food baskets for twenty-six European countries; second, we priced them using national consumer price index data. The advantage of Linear Programming is that both specific consumption habits, that is, the social acceptance of the food basket, and age- and gender-specific dietary needs can be considered at the same time. However, there is a trade-off between social acceptance and healthiness of the diets, which can be addressed by Linear Programming in a transparent manner, as the method is objectively comprehensible and adaptable. Additionally, the method is based on official, representative data and allows for easy recalculation when new consumption and price data become available.

Previous methods for determining European Food Reference Budgets relied predominantly on methods in which nutrition experts ensured the health aspect and focus group discussions social acceptance^(e.g.(8,10–15))^. This proceeding was criticised in the literature as the outcome is dependent on the respective composition of the focus groups so that objectivity and transparency could not necessarily be guaranteed^(17,46)^. Another method for determining Food Reference Budgets is to derive food consumption from the behaviour of lower income groups observed in national surveys (e.g. calculation of social assistance in Germany). Although this procedure fulfils the claim of objectivity and transparency, on the one hand, health aspects remain unconsidered and on the other hand consumption behaviour may be influenced by the financial constraints of the lower income groups which bears the risk of circularity.

Using Linear Programming for Food Reference Budgets produced plausible results: On the one hand, positive correlations between observed and optimised consumption showed that country specific consumption habits could be considered. On the other hand, deviations between observed and optimised consumption highlighted necessary adjustments, such as increasing vegetable intake and reducing sweets and snacks to meet dietary recommendations. These adjustments seem to be necessary against the background of current dietary recommendations. If full implementation of all dietary recommendations is considered incompatible with social acceptability, the model allows for transparent adjustments. We carried out the calculations in this study exemplarily for men and women aged 24–49 years as an example. Since the requirements for a healthy diet differ according to age- and gender groups, the results for other groups are different from those presented here. However, all age- and gender-specific consumption habits and dietary recommendations could be easily implemented in the Linear Programming model, provided that appropriate data are available.

The accuracy of Linear Programming results depends on input data quality. To consider country-specific consumption habits, we used the EU-HBS. Although the EU-HBS is based on a standardised food classification system, the survey methods vary between countries: some countries use one or more interviews, others use diaries, highlighting the need for standardised data collection across the EU.

Another limitation of the data is that the classification system is primarily commodity based. For example, the ECOICOP food classification used in the EU-HBS does not differentiate between wholegrain and refined products within categories such as rice, pasta or bread. As a result, the potential differences in nutritional quality between these subtypes could not be reflected in the model.

Moreover, the data do not include home-grown or gifted foods, food consumed outside the home (e.g. in restaurants or canteens) or food waste. In Germany, for example, approximately 28 % of total food expenditure is allocated to out-of-home consumption^(47)^. Given the higher cost of such food, its quantitative share is likely considerably lower, so that at least in Germany most of the food consumption takes place at home. While the exclusion of out-of-home consumption may lead to an underestimation of food quantities, the absence of information on food waste may have the opposite effect and result in overestimation.

Food consumption surveys could provide an alternative to consumption survey data; however, they are subject to limitations such as under- or over-reporting of specific food groups (e.g. vegetables or confectionery). Moreover, harmonised food consumption data across all twenty-six European countries were not available. For the objective of this study – to evaluate the suitability of Linear Programming for the calculation of Food Reference Budgets – the EU-HBS provided a sufficiently consistent and representative data basis. Nevertheless, future applications aiming at more detailed nutritional modelling would benefit from datasets that allow for a finer differentiation of food characteristics.

Another limitation of the data is that we only had average consumption values from the EU HBS, which did not allow us to set consumption limits based on the observed percentiles. Whilst a fixed lower limit (10 % of average intake) was introduced to maintain diversity, it was not possible to apply a uniform upper limit due to the different consumption patterns in individual countries. Future applications should include the percentiles of intake when available to improve the validity of the results.

Each food group was assigned an average nutrient composition based on the German food composition database (BLS). Where a direct match was not possible, the average of multiple representative food items was used. While this approach cannot fully reflect the nutritional variation within broader food groups, it was necessary due to the aggregated nature of the EU-HBS data. For future applications, using more disaggregated data with item-level nutrient values would allow for greater precision in modelling dietary quality.

For dietary recommendations, we relied on the values of the EFSA but supplemented them with WHO and German Nutrition Society guidelines for missing food-specific recommendations. Since food related-recommendations are considered important^(48)^, they should be published by EFSA for the most important foods.

The food baskets identified by Linear Programming were priced in a second step using consumer price index data, which were only available for Germany. The prices for the other countries were derived using Eurostat-Price level indices. Both the German consumer price index data and the Eurostat-Price level indices are representative datasets from the official statistics. In previous studies to determine European Food Reference Budgets, prices were collected by a single observer at a specific time in only a few shops so that representativeness and reliability could not be guaranteed^(49)^. Although the price data used here represent a significant improvement over previous studies, the use of consumer price index data from all EU countries would be desirable for future studies.

As an illustrative comparison, the study by Pedroni *et al.* (2024) provides a useful benchmark for assessing the plausibility of our results. Using 2014 price and consumption data for Belgium, they estimated daily costs of a healthy diet at €5·60 for women and €6·29 for men^(50)^. Our estimates for Belgium, based on 2018 prices, ranged from €4·71 to €7·87 depending on the price percentile level applied. Despite methodological and temporal differences, the similar cost range suggests that our approach provides a reasonable basis for estimating the cost of a healthy diet.

The results have shown that Food Reference Budgets vary between countries, but to a lesser extent than mean net income. This has also been shown in other studies^(49)^. As a result, in some countries, comparatively high shares of income would have to be spent on a healthy diet. Comparing the cost of a healthy diet relative to income is therefore particularly important. Dietary patterns that appear inexpensive in absolute terms – such as in Poland, where the cost of a healthy diet amounts to €2·40 for women and €2·38 for men per day (evaluated at the 20th price percentile) – represent a significantly higher share of disposable income, at just over 13 %. In contrast, countries with higher food costs but disproportionately higher incomes (e.g. Luxembourg or Germany) show lower relative expenditure shares. This indicates that affordability is not solely a question of food prices but must be interpreted in the context of overall income levels. This also indicates that in some countries it will be much more challenging to meet food security targets. However, some authors stress that food insecurity should not be considered as an isolated problem. Rather, food policies should be integrated in social and economic policies to face inequality and ensure adequate income sufficient for healthy diets^(3)^.

Since there is no universally accepted threshold that defines when food becomes unaffordable, we refrained from applying a fixed cut-off value. Instead, the cost of the modelled healthy diets was expressed as a share of national median disposable income, allowing for cross-country comparability while leaving room for contextual interpretation by national stakeholders.

As the focus was on minimum costs, we used lower price percentiles for the valuation of the food baskets. Since it is unclear which lower price levels are actually feasibly in practice, we valued the baskets with different lower price percentile values ranging from the 20th to the 50th. In order to determine the minimum price level that can be actually implemented in practice, research should be carried out in this regard.

Overall, this study has shown that Linear Programming could be a promising approach for determining uniform and comparable European Food Reference Budgets and should therefore be discussed in the context of the EU Commission’s efforts to modernise the European minimum income schemes.

## Supporting information

Taeger and Thiele supplementary material Taeger and Thiele supplementary material

## Data Availability

All datasets used in this study are part of the official statistics of the countries and/or the EU and have been used under licence, so we are not allowed to distribute them. However, it is possible to request the data from the respective institutions.

## Supplementary material

For supplementary material accompanying this paper visit https://doi.org/10.1017/S1368980025101316
