# Supplementary material for: How expensive is a healthy diet in Europe? Using Linear Programming as a standardised method for calculating European Food Reference Budgets
Source: Public Health Nutr. 2025 Nov 3;29(1):e1. doi: 10.1017/S1368980025101316 (PMC12809604; doi:10.1017/S1368980025101316)
Supplement: Taeger and Thiele supplementary material [file S1368980025101316sup001.docx]

# Annex

***Annex 1****: Food Reference Budgets – costs for a healthy diet in € per day for different price percentiles*

| **Country** | **Food Reference Budgets (Cost of healthy diet) [€/day]** | | | | | | | |
| --- | --- | --- | --- | --- | --- | --- | --- | --- |
|  | **Woman** | | | | **Man** | | | |
|  | 50th price percentile | 40th price percentile | 30th price percentile | 20th price percentile | 50th price percentile | 40th price percentile | 30th price percentile | 20th price percentile |
| Luxembourg | 8.28 | 7.27 | 6.47 | 5.57 | 8.23 | 7.03 | 6.10 | 5.28 |
| Italy | 7.59 | 6.48 | 5.73 | 5.00 | 7.14 | 6.14 | 5.33 | 4.58 |
| Belgium | 7.47 | 6.53 | 5.55 | 4.71 | 7.87 | 6.78 | 5.88 | 4.98 |
| France | 7.36 | 6.36 | 5.70 | 5.00 | 7.43 | 6.43 | 5.77 | 4.97 |
| Austria | 7.20 | 5.87 | 5.06 | 4.40 | 8.51 | 7.32 | 6.40 | 5.58 |
| Ireland | 6.99 | 5.98 | 5.34 | 4.58 | 7.61 | 6.52 | 5.61 | 4.78 |
| Denmark | 6.86 | 5.69 | 4.90 | 4.27 | 8.70 | 7.47 | 6.54 | 5.64 |
| Malta | 6.58 | 5.57 | 4.87 | 4.27 | 6.67 | 5.69 | 4.91 | 4.19 |
| Sweden | 6.48 | 5.68 | 5.03 | 4.24 | 6.98 | 6.12 | 5.51 | 4.67 |
| Cyprus | 6.37 | 5.33 | 4.69 | 4.07 | 6.45 | 5.58 | 4.94 | 4.30 |
| The Netherlands | 6.30 | 5.33 | 4.68 | 4.06 | 7.19 | 6.16 | 5.40 | 4.71 |
| Portugal | 6.23 | 5.50 | 4.74 | 4.02 | 5.86 | 5.00 | 4.33 | 3.75 |
| Spain | 6.16 | 5.25 | 4.65 | 3.95 | 5.19 | 4.39 | 3.90 | 3.36 |
| Greece | 6.05 | 4.85 | 4.21 | 3.69 | 6.11 | 5.21 | 4.56 | 3.97 |
| Finland | 5.92 | 4.91 | 4.25 | 3.67 | 8.84 | 7.67 | 6.70 | 5.71 |
| Slovenia | 5.78 | 4.99 | 4.47 | 3.79 | 5.92 | 5.07 | 4.42 | 3.83 |
| Latvia | 5.69 | 4.86 | 4.39 | 3.67 | 5.40 | 4.65 | 4.04 | 3.48 |
| Germany | 5.50 | 4.39 | 3.77 | 3.30 | 6.40 | 5.46 | 4.76 | 4.10 |
| Slovakia | 5.23 | 4.30 | 3.72 | 3.19 | 5.15 | 4.39 | 3.80 | 3.25 |
| Estonia | 5.20 | 4.39 | 3.88 | 3.29 | 6.02 | 5.18 | 4.50 | 3.84 |
| Lithuania | 4.72 | 3.99 | 3.61 | 3.00 | 4.78 | 4.10 | 3.55 | 3.02 |
| Romania | 4.22 | 3.66 | 3.30 | 2.80 | 3.66 | 3.13 | 2.78 | 2.42 |
| Czech Republic | 4.19 | 3.32 | 2.84 | 2.48 | 4.57 | 3.87 | 3.34 | 2.91 |
| Hungary | 4.17 | 3.37 | 2.93 | 2.54 | 5.12 | 4.38 | 3.83 | 3.35 |
| Poland | 3.94 | 3.24 | 2.82 | 2.40 | 3.73 | 3.20 | 2.78 | 2.38 |

| **Country** | **%-share of Food Reference Budgets of income** | | | | **Change between the 50th and 20th price percentile [%]** |
| --- | --- | --- | --- | --- | --- |
|  | **50th price percentile)** | **40th price percentile)** | **30th price percentile)** | **20th price percentile)** |  |
| Luxembourg | 8.74 | 7.57 | 6.65 | 5.74 | 3.00 |
| Denmark | 9.43 | 7.98 | 6.93 | 6.01 | 3.43 |
| Germany | 9.56 | 7.92 | 6.86 | 5.95 | 3.61 |
| Sweden | 9.62 | 8.43 | 7.53 | 6.36 | 3.25 |
| The Netherlands | 10.25 | 8.74 | 7.66 | 6.67 | 3.58 |
| Ireland | 10.69 | 9.15 | 8.02 | 6.86 | 3.83 |
| Finland | 10.98 | 9.35 | 8.14 | 6.98 | 4.00 |
| Austria | 11.39 | 9.56 | 8.30 | 7.24 | 4.15 |
| Belgium | 11.82 | 10.25 | 8.80 | 7.47 | 4.35 |
| France | 12.15 | 10.50 | 9.42 | 8.18 | 3.97 |
| Spain | 14.00 | 11.90 | 10.55 | 9.02 | 4.99 |
| Cyprus | 15.26 | 12.98 | 11.47 | 9.95 | 5.31 |
| Italy | 15.95 | 13.68 | 11.98 | 10.38 | 5.57 |
| Slovenia | 16.12 | 13.86 | 12.24 | 10.50 | 5.62 |
| Malta | 16.36 | 13.91 | 12.08 | 10.45 | 5.91 |
| Czech Republic | 17.60 | 14.44 | 12.41 | 10.84 | 6.76 |
| Estonia | 19.47 | 16.58 | 14.54 | 12.37 | 7.11 |
| Poland | 21.30 | 17.87 | 15.53 | 13.25 | 8.05 |
| Portugal | 23.61 | 20.50 | 17.70 | 15.18 | 8.43 |
| Lithuania | 25.14 | 21.41 | 18.95 | 15.95 | 9.19 |
| Slovakia | 25.37 | 21.26 | 18.40 | 15.75 | 9.62 |
| Latvia | 27.60 | 23.65 | 20.97 | 17.80 | 9.80 |
| Greece | 28.22 | 23.36 | 20.36 | 17.76 | 10.45 |
| Hungary | 31.24 | 26.08 | 22.72 | 19.84 | 11.40 |
| Romania | 43.76 | 37.73 | 33.81 | 29.00 | 14.76 |

***Annex 2****: Share of Food Reference Budgets of median equivalence total net income 2018*
